# The adverse drug reaction reporting assignment for specialist oncology nurses: a preliminary evaluation of quality, relevance and educational value in a prospective cohort study

**DOI:** 10.1007/s00210-017-1430-z

**Published:** 2017-10-23

**Authors:** Tim Schutte, Rike van Eekeren, Milan Richir, Jojanneke van Staveren, Eugène van Puijenbroek, Jelle Tichelaar, Michiel van Agtmael

**Affiliations:** 1 Department of Internal Medicine, Pharmacotherapy Section, VU University Medical Center, Room ZH4A50, De Boelelaan 1117, 1081 HZ Amsterdam, The Netherlands; 2 RECIPE (Research & Expertise Center In Pharmacotherapy Education), Amsterdam, The Netherlands; 3 Department of Pharmacy, Pharmacotherapy and Pharmaceutical Care, University of Groningen, Groningen, The Netherlands; 4 WHO Collaborating Centre for Pharmacovigilance in Education and Patient Reporting, ‘s-Hertogenbosch, The Netherlands; 5 The Netherlands Pharmacovigilance Centre Lareb, ‘s-Hertogenbosch, The Netherlands; 6 Amstel Academy, VU University Medical Center, Amsterdam, The Netherlands

**Keywords:** Oncology nurses, Nursing education, Pharmacotherapy, Pharmacovigilance, ADR reporting

## Abstract

In a new prescribing qualification course for specialist oncology nurses, we thought that it is important to emphasize pharmacovigilance and adverse drug reaction (ADR) reporting. We aimed to develop and evaluate an ADR reporting assignment for specialist oncology nurses. The quality of report documentation was assessed with the “Clinical Documentation tool to assess Individual Case Safety Reports” (ClinDoc). The relevance of the reports was evaluated in terms of ADR seriousness, the listing for additional monitoring of the drug by European Medicines Agency (EMA), and lack of labelling information about the ADR. Nurses’ opinions of the assignment were evaluated using an E-survey. Thirty-three ADRs were reported, 32 (97%) of which were well documented according to ClinDoc. Thirteen ADRs (39%) were “serious” according to CIOMS criteria. In five cases (15%), the suspect drugs were listed for additional monitoring by EMA and in seven cases (21%), the ADR was not mentioned in the Summary of Product Characteristics. Twenty-five (78.1%) of the 32 enrolled nurses completed the E-survey. Most were > 45 years of age (68%), female (92%) and had extensive clinical experience (6–33 years). All agreed or completely agreed that the reporting assignment was useful, that it fitted in daily practice and that it increased their attention for medication/patient safety. A large majority (84.0%) agreed the assignment changed how they dealt with ADRs. Specialist oncology nurses are capable of reporting ADRs, and they considered the assignment useful. The assignment yielded valuable, relevant, and well-documented ADR reports for pharmacovigilance practice.

**Electronic supplementary material:**

The online version of this article (10.1007/s00210-017-1430-z) contains supplementary material, which is available to authorized users.

## Introduction

The spontaneous reporting of adverse drug reactions (ADRs) by health professionals is a widely used method for ADR detection (Miguel et al. 2013). This is vital for identifying unknown, uncommon and serious ADRs, with a view to improving medication safety and understanding the risks of drugs (Molokhia et al. 2009; Sultana et al. 2013). However, this spontaneous reporting system is dependent on the responsiveness of health professionals and on the quality and quantity of their ADR reports. Reporting ADRs is typically the responsibility of physicians and is mandatory in some countries such as the Netherlands (Hazell and Shakir 2006; Lopez-Gonzalez et al. 2009; Molokhia et al. 2009). Nevertheless, underreporting remains a barrier to optimal ADR monitoring (Hazell and Shakir 2006; Lopez-Gonzalez et al. 2009; Molokhia et al. 2009). To stimulate ADR reporting, pharmacists, medical/pharmacy students, patients, and nurses are now authorized to report ADRs, which has the added advantage of obtaining information from other, non-physician sources (van Grootheest et al. 2003; Steurbaut and Hanssens 2014; van Eekeren et al. 2014; Harmark et al. 2015).

Nurses are a potentially valuable source of ADR reports, because they administer most drugs in hospitals and are often present when an ADR occurs (Hall et al. 1995). Furthermore, nurses report different types of suspected ADRs from those reported by physicians; for instance, they report more side effects after parenteral administration (Hall et al. 1995; Sacilotto et al. 1995; Ranganathan et al. 2003; Bigi and Bocci 2017). Although nurse reporting seems promising, it has been queried whether nurses are adequately prepared for this role. Previous studies have shown that they have little knowledge and poor practice regarding pharmacovigilance and the spontaneous reporting system (Hanafi et al. 2012; Hanrath 2016; Salk and Ehrenpreis 2016). Moreover, a recent literature review emphasized the need for pharmacovigilance training in (postgraduate) nurse education (Bigi and Bocci 2017).

When we developed a new prescribing qualification course for specialist oncology nurses, we wanted to focus attention on their role in drug safety. Although this prescribing qualification allows nurses to prescribe a limited set of drugs, their role in pharmacovigilance would cover the entire field of oncology, with its multiple drugs, many of which give rise to serious ADRs. It is unclear how to best prepare (specialist) nurses for this task. Some interventions have been shown to be effective for qualified physicians, but few studies have investigated other health professionals receiving further training (Pagotto et al. 2013). While passive educational methods (e.g. lectures) are typically used during training (Rosebraugh et al. 2003; Durrieu et al. 2007), those being taught prefer active learning forms (Elkalmi et al. 2011; Gavaza and Bui 2012; Schutte et al. 2017c). Such active learning approaches are preferential for adult learners (Yardley et al. 2012). By combining the preference for an active learning approach and our experience with a student ADR-reporting assignment (van Eekeren et al. 2014; van Eekeren and Schutte 2015), we hypothesized that an ADR reporting assignment would be a suitable approach for training the pharmacovigilance skills of specialist oncology nurses.

Therefore, the primary objective of this study was to establish the value of an ADR reporting assignment to pharmacovigilance centres and specialist nurses following a prescribing training course. A secondary objective was to evaluate the preparedness of these specialist nurses for their role in pharmacovigilance, their intention/attitudes and skills/ towards pharmacovigilance and ADR reporting.

## Methods

### Setting

**Fig. 1:**
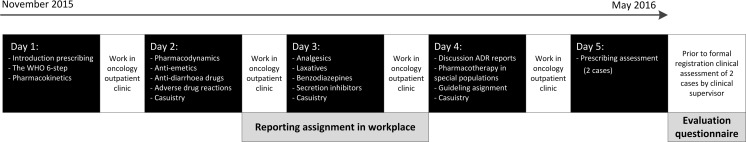
Overview of the first course on prescribing at the Amstel Academy (VU University Medical Center) for specialist oncology nurses between November 2015 and May 2016. The nurses follow the course in addition to their work in different (most non-academic) hospitals in the Netherlands which is depicted in the white boxes. The course consists of 4 days (6 h/day) equally spread over half a year, which is depicted as black boxes. Within these boxes, the discussed themes are noted. The nurses were instructed regarding the reporting assignment during a lecture in day 2 (January). This lecture included a general lecture on pharmacovigilance in the Netherlands. The ADRs had to be reported before the group discussion (day 4, in March). Following the last day of the module, the evaluation survey was distributed

The Amstel Academy (VU University Medical Center) offers registered specialist oncology nurses a course on prescribing to enable them to qualify to prescribe a limited set of frequently prescribed drugs (anti-diarrhoea drugs, anti-emetics, analgesics (non-opioids) and benzodiazepines). Nurses follow the course in addition to their work in different (most non-academic) hospitals in the Netherlands. The course consists of 4 days (6 h/day) spread over half a year, completed by a prescribing assessment. The module overview is displayed in Fig. 1. It covers pharmacovigilance by means of a lecture on pharmacovigilance and a practical ADR reporting assignment. The reporting assignment was introduced during a pharmacovigilance lecture, in which the nurses were instructed to report an ADR that was either unknown, exceptional or unexpected to them. The assignment was followed by a group discussion of the ADRs reported, led by a pharmacotherapy teacher (T.S.) and assessor from the Pharmacovigilance Centre Lareb (R.vE.).

### Population

Thirty-two specialist oncology nurses enrolled for this course in November 2015 in two separate groups. All were invited to voluntarily participate in this study and complete an anonymous E-questionnaire after the course. Based on previous E-questionnaire studies, we expected a response rate of 25–50%.

### Instruments

Two aspects of the course were evaluated. First, the quality of documentation and relevance of the ADRs reported and second, the nurses’ perspective of the reporting assignment together with their current attitudes and skills regarding pharmacovigilance and ADR reporting.

The quality of documentation of the reported ADRs was measured by an assessor from the Pharmacovigilance Centre Lareb, using the novel “Clinical Documentation tool to assess Individual Case Safety Reports” (ClinDoc). The ClinDoc tool was previously described by Rolfes and provides a completeness score (0–100%) (Rolfes et al. 2016a; Oosterhuis et al. 2016; Rolfes et al. 2016b; Rolfes et al. 2017). It assesses the relevance of the information provided in an ADR report (e.g. information on the ADR, time relationship, drug, and patient characteristics). The ClinDoc is displayed in Table 1.

**Table 1:** The ClinDoc tool (clinical documentation score), as it is developed by Lareb as part of WEB-RADR (WEB-RADR 2017; Rolfes et al. 2017). The ClinDoc tool comprises four domains: (1) description of the ADR, (2) description of the chronology of the ADR, (3) suspected drug and (4) patient characteristics. These domains consist of multiple subdomains. Firstly, an Individual Case Safety Reports (ICSRs) can be scored using this tool by first identifying which subdomains are relevant. Secondly, the assessor indicates if this relevant information was present or absent. Domain scores are calculated by dividing the score for present information by the number of subdomains deemed relevant. The final score is the mean of the domain scores. This final score can be categorised into three categories of reporting completeness, being well (≥ 75%), moderately (45–75%) or poorly (≤ 45%)

The ClinDoc tool				
		Relevant? Yes (+ 1) No (n.a.)	Present? Yes (+ 1) No (0)	Domain score
1: Description of the adverse drug reaction (ADR)				
a	Proper description of the ADR			
b	Specification complaints “localization” and “characterization” To strengthen the diagnosis (item c or d or e if applicable):			
c	Treatment; *or*			
d	Visual material (photo, video); *or*			
e	Lab values, test			
2: Description of the chronology				
a	Latency			
b	Description of the course of the ADR			
c	Action taken on drug			
d	Outcome of the ADR			
3: Suspected drug				
a	Brand name in case of drug substitution?			
b	Different forms or route of administration for suspected drug?			
c	Dose-relationship with ADR?			
d	Batch number of relevance?			
4: Patient characteristics				
a	Risk factors/medical history/comorbidity/indication			
b	Concomitant medication			
c	Age/gender/length/weight			
d	Patient’s lifestyle or other risk factors			

The relevance of the reported ADR was assessed with respect to label information for the suspect drug (in Summary of Product Characteristics), seriousness of the ADR (According to CIOMS criteria 1999), additional monitoring of the drug, off-label use of the drug and severity of the ADR as a reason to stop necessary treatment. The nurses were not informed that their reports would be assessed for completeness.

The nurses’ perspective was evaluated with an E-questionnaire covering three themes (*intention/attitudes*, *knowledge/skills and evaluation of pharmacovigilance teaching*) in 13 questions. Participants were sent an information letter and returned an informed consent statement before they completed the questionnaire. They also provided information about baseline characteristics, including their earlier ADR reporting experience and whether ADR reporting was covered in their initial training as nurse. In the E-questionnaire, once a question was answered, it was not possible for respondents to return to earlier answers (since some questions consisted of the answers to earlier questions). There was no time limit for E-questionnaire completion, but on the basis of a pilot study, we estimated that it would take 8–10 min to complete. The first two themes *intention/attitudes and knowledge/skills* regarding pharmacovigilance and ADR reporting were investigated using a set of open-ended question and dichotomous questions that were used in earlier studies (Schutte et al. 2017c; Schutte et al. 2017d). The third theme was an evaluation of the ADR-reporting assignment and (previous) pharmacovigilance teaching. This theme consisted of the open question “what have you learned?” and 18 statements that covered participants’ opinions of the ADR-reporting assignment, discussion of the ADR-reporting assignment, their current and past education in pharmacovigilance and whether they considered this education sufficient and appropriate for future clinical practice. Answers were scored on a Likert scale (5- or 7-point). The complete questionnaire is displayed in Appendix 1.

### Data analysis

All data were imported in SPSS Statistics 22 (IBM Corp.; Armonk, New York). Descriptive statistics were used to report frequencies and means/standard deviations (SD) of survey results. Open questions were analysed using content/thematic analysis (Braun and Clarke 2006). The mean composite knowledge score was calculated as the sum of the correct answers divided by the number of questions answered (uncorrected for guessing). Skills were analysed as two separate outcomes (i.e. knowing where to report and knowing what to report). A significance level with an alpha of 5% was considered statistically significant (*p* < 0.05) in all analyses.

## Results

All 32 oncology nurses enrolled in the prescribing qualification course were invited to participate in this study by e-mail; 25 completed the questionnaire, yielding a response rate of 78.1%. Of the responders, 23 were female (92%), 22 (68%) were 45 years or older and their clinical experience as nurse ranged between 6 and 33 years. While 19 nurses (76%) reported that their initial training curriculum covered ADRs, only 2 nurses (8%) indicated that the curricula covered the reporting of ADRs. Before they enrolled, 7 nurses (28%) had reported one or more ADRs to the Pharmacovigilance Centre Lareb.

### Quality and relevance of reports

A total of 33 Individual Case Safety Reports (ICSRs) were reported during the assignment, accounting for 41 ADRs. In 23 (70%) of the ICSRs, the suspect drug was a cytostatic agent, used in the treatment of malignant diseases. Gastrointestinal disorders and skin reactions were the most frequently reported ADRs. Overall, 32 (97%) of the reports were well documented. Most ICSRs were considered relevant, in terms of seriousness (according to CIOMS) of the ADR (*n* = 13, 39%), lack of label information about the reported ADR (*n* = 7, 21%), additional monitoring of the suspect drug by EMA (European Medicine Agency; *n* = 4, 12%), the ADR being the cause of withdrawal of a cytostatic drug (*n* = 4, 12%) and off-label use of the suspect drug (*n* = 2, 6%). Eleven (33%) reports used hospitalization as criterion of ADR seriousness, one used a life-threatening situation and one used death. Further details regarding ICSRs, ClinDoc score and relevance are displayed in Table 2 and Appendix 1.

**Table 2:** Information about characteristics, clinical documentation score and relevance of Individual Case Safety Reports, reported by specialist oncology nurses and assessed by the pharmacovigilance assessors using the Clinical documentation (ClinDoc) tool (see Table 1)

Individual Case Safety Reports	
Number of Individual Case Safety Reports	*n* = 33
Number of reported ADRs (grouped by System Organ Class)	*n* = 41
Clinical documentation score (overall) (%)	
Mean (range)	89% (61–100%)
Median	92%
Clinical documentation score, domain 1	
Mean (range)	84% (50–100%)
Median	100%
Clinical documentation score, domain 2	
Mean (range)	88% (67–100%)
Median	100%
Clinical documentation score, domain 3	
Mean (range)	100% (100–100%)
Median	100%
Clinical documentation score, domain 4	
Mean (range)	93% (33–100%)
Median	100%
Type of drug	
Cytostatic	*n* = 23 (70%)
Supporting	*n* = 7 (21%)
Diagnostic	*n* = 1 (3%)
Other treatment	*n* = 2 (6%)
System Organ Class of reported ADRs (%)	
Gastrointestinal disorders	*n* = 10 (30%)
Skin and subcutaneous tissue disorders	*n* = 10 (30%)
Respiratory, thoracic and mediastinal disorders	*n* = 4 (12%)
Eye disorders	*n* = 3 (9%)
Nervous system disorders	*n* = 3 (9%)
Psychiatric disorders	*n* = 2 (6%)
Blood and lymphatic system disorders	*n* = 1 (3%)
Cardiac disorders	*n* = 1 (3%)
Drug interaction	*n* = 1 (3%)
General disorders and administration site conditions	*n* = 1 (3%)
Hepatobiliary disorders	*n* = 1 (3%)
Immune system disorders	*n* = 1 (3%)
Musculoskeletal disorders	*n* = 1 (3%)
Renal and urinary disorders	*n* = 1 (3%)
Reproductive and breast disorders	*n* = 1 (3%)
Relevance of reported ICSR (%)	
Seriousness	*n* = 13 (39%)
ADR not labelled	*n* = 7 (21%)
Additional monitoring of drug	*n* = 5 (15%)
ADR cause of withdrawal of oncolytic therapy	*n* = 4 (12%)
Off-label use of drug	*n* = 2 (6%)

### The ADR-reporting assignment and pharmacovigilance teaching

**Fig. 2:**
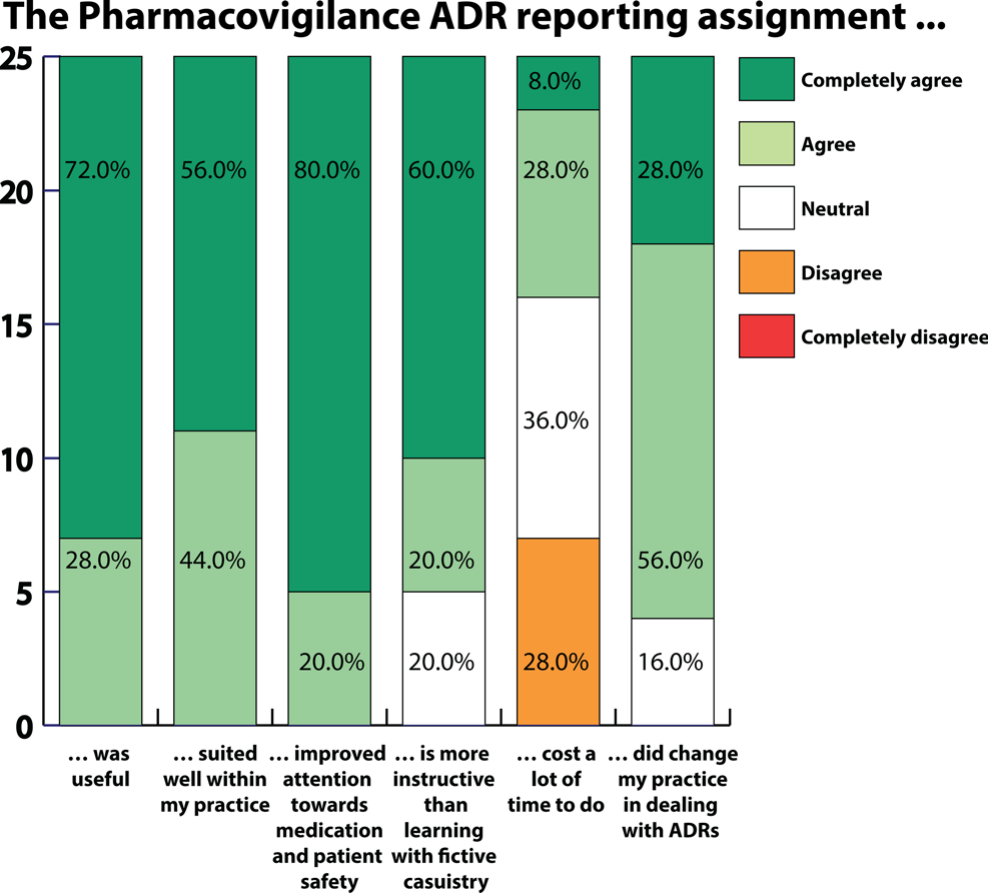
Results of evaluation (on a 5-point Likert scale) of a pharmacovigilance reporting assignment, part of a prescribing qualification course for specialist oncology nurses, and discussion of reported adverse drug reactions (ADRs)

The 25 participants agreed that the reporting assignment was useful, that it was consistent with their daily work and duties and that it made them more aware of medication and patient safety. Only nine participants (36.0%) thought that the reporting assignment cost a lot of time. Twenty-one participants (84.0%) agreed that the reporting assignment changed how they dealt with ADRs. The results of the participant evaluation are displayed in Fig. 2.

### Intention/attitudes

After participation, all the nurses reported they intended to report serious and unknown ADRs, because reporting them “contributed to medication safety” and “improved patient safety”. They also considered that it was “personally beneficial” and “educated others about drug risks”. The nurses did not consider that reporting ADRs would “break trust with patients” or that it would “increase the risk of malpractice”. The nurses’ attitudes towards ADR reporting in different situations are displayed in Table 3.

**Table 3:** Intention, attitudes and opinions regarding the reporting of adverse drug reactions (ADR) and pharmacovigilance (education) among specialist nurses after the ADR-reporting assignment

Intention, attitudes and opinions about adverse drug reactions (ADR) reporting and pharmacovigilance (education)									
	*N*	Mean (SD)	1—Extremely unlikely	2	3	4—Neither likely nor unlikely	5	6	7—Extremely likely
Could you indicate how likely it is you will report an ADR to Lareb in the following situations:									
I intend to report all ADRs that I will encounter to the competent authority	25	5.92 (1.2)	–	1 (4.0%)	–	2 (8.0%)	2 (8.0%)	12 (48.0%)	8 (32.0%)
I intend to report unknown ADRs that I will encounter to the competent authority	25	6.36 (0.7)	–	–	–	–	3 (12.0%)	10 (40.0%)	12 (48.0%)
I intend to report serious ADRs that I will encounter to the competent authority	25	6.64 (0.6)	–	–	–	–	1 (4.0%)	7 (28.0%)	17 (68.0%)
How likely do you think the following outcomes will be if you report a serious ADR:									
It contributes to the safe use of medicines.	25	6.6 (0.5)	–	–	–	–	–	9 (36.0%)	16 (64.0%)
Improves patient safety	25	6.5 (0.5)	–	–	–	–	–	12 (48.0%)	13 (52.0%)
Educates others about drug risks	25	6.1 (0.8)	–	–	–	1 (4.0%)	4 (16.0%)	12 (48.0%)	8 (32.0%)
Personally beneficial	25	6.2 (0.6)	–	–	–	–	2 (8.0%)	16 (64.0%)	7 (28.0%)
Time consuming to report	25	4.5 (1.6)	–	5 (20.0%)	–	7 (28.0%)	6 (24.0%)	5 (20.0%)	2 (8.0%)
Disrupts the normal workflow	25	4.0 (1.8)	3 (12.0%)	3 (12.0%)	3 (12.0%)	6 (24.0%)	4 (16.0%)	4 (16.0%)	2 (8.0%)
Increases risk of malpractice	25	2.6 (1.6)	8 (32.0%)	7 (28.0%)	4 (16.0%)	3 (12.0%)	–	3 (12.0%)	–
Breaks trust with patients	25	1.9 (1.3)	12 (48.0%)	9 (36.0%)	2 (8.0%)	1 (4.0%)	–	0	1 (4.0%)
			*N*	Mean (SD)	Completely disagree	Disagree	Neutral	Agree	Completely agree
Opinion regarding (current) education in pharmacovigilance									
Pharmacovigilance should be included as a core topic in the curriculum of all prescribers			25	4.4 (0.7)	–	1 (4.0%)	–	11 (44.0%)	13 (52.0%)
Pharmacovigilance is well covered (up to now) in my curriculum			25	3.6 (1.0)	–	5 (20.0%)	5 (20.0%)	11 (44.0%)	4 (16.0%)
I do not know how I should report an ADR to the relevant authorities			25	1.4 (0.6)	16 (64.0%)	8 (32.0%)	1 (4.0%)	–	–
Opinion regarding current and future role in pharmacovigilance									
Students can report ADRs during their clerk/internships			25	4.0 (1.0)	1 (4.0%)	1 (4.0%)	2 (8.0%)	13 (52.0%)	8 (32.0%)
Reporting known ADRs makes no significant contribution to the reporting system.			25	2.0 (0.8)	7 (28.0%)	13 (52.0%)	4 (16.0%)	1 (4.0%)	–
With my present knowledge, I am very well prepared to report any ADRs in my future practice.			25	4.4 (0.7)	–	1 (4.0%)	–	13 (52.0%)	11 (44.0%)
I believe that doctors are one of the most important healthcare professionals to report ADRs			25	3.5 (1.0)	1 (4.0%)	3 (12.0%)	7 (28.0%)	10 (40.0%)	4 (16.0%)
I believe that pharmacists are one of the most important healthcare professionals to report ADRs			25	3.3 (1.1)	1 (4.0%)	5 (20.0%)	8 (32.0%)	8 (32.0%)	3 (12.0%)
I believe that (specialist) nurses are one of the most important healthcare professionals to report ADRs			25	3.8 (0.7)	–	–	8 (32.0%)	13 (52.0%)	4 (16.0%)
I believe that serious and unexpected reactions that are not fatal or life-threatening during clinical trials should not be reported.			25	1.2 (0.4)	19 (76.0%)	6 (24.0%)	–	–	–

### Knowledge and skills

After the reporting assignment, all 25 nurses who returned the questionnaire said they knew where to report ADRs, and all but one (96%) knew what information was needed to fill in an ADR report. The mean score for the 12 knowledge questions was 75% (SD 13). The results for the individual questions are displayed in Table 4. Analysis of the open-ended questions showed that nurses considered that they had learned why it is important to be aware of ADRs and how to recognize them, that they had experienced and learned how and what to report and that they had learned more about the Pharmacovigilance Centre Lareb and its role in assessing ADRs, in providing feedback, and as knowledge centre for drug safety (Table 4).

**Table 4:** Knowledge/skills regarding the reporting of adverse drug reactions and open feedback on what the specialist oncology nurses perceived they had learned (some quotes/examples fit into more than one theme)

Skills and knowledge of reporting adverse drug reactions (ADRs) and pharmacovigilance		
		Total
Students who did not know where to report an ADR		0
Students who did not know which items were necessary for a good ADR report		1 (4%)
		% with correct answer
1. All ADRs, irrespective of severity, must be reported (*no)		36
2. Doctors should report serious ADRs even if uncertain that product caused the event (*yes)		100
3. Doctors should report serious ADRs even if do not have all details of event (*yes)		92
4. All serious ADRs are known before a drug is marketed (*no)		84
5. Lareb does not disclose ADR reporter’s identity (*yes)		84
6. One can report ADRs anonymously to Lareb (*yes)		76
7. Adverse experiences with cosmetics and special nutritional products may be reported to Lareb (*yes)		28
8. Adverse experiences with natural or homeopathic products may be reported to Lareb (*yes)		60
9. Adverse experiences with vaccines may be reported to Lareb (*yes)		100
10. One case reported by a doctor does not contribute much to knowledge about drug risks (*no)		72
11. I have adequate knowledge of ADR reporting (*yes)		92
12. Patients can report ADRs independent from a healthcare professional (*yes)		80
Total 12 knowledge questions		75 (SD 13)
Qualitative results—student reflections of what they learned		
Themes		Examples
Adverse drug reactions		
	Awareness	Being more aware of adverse drug reactions, no longer the attitude “that’s part of the job”.
		Now, I have a different perspective on all drugs patients use, I am aware of possible interactions and side effects of drugs.
	Recognizing	Figuring out which drug could have caused the adverse drug reaction was very useful, and also to search for alternative causes underlying the adverse drug reaction.
		Recognizing adverse drug reactions.
		It was very instructive to search for the drug that could have caused a certain adverse drug reaction.
Reporting		
	How to report/experience	Where and how to report an adverse drug reaction. Because you make a report yourself, you learn at the same time. The assignment was good and fits well together with my daily work and duties.
		Being well prepared to report the adverse drug reaction, there are many queries in the report form. I first read the entire report form to prepare myself.
	What information to collect and report	Which items are necessary for an ADR-report.
		Figuring out properly and accurately what the complaints are. Making sure I collect all information necessary.
	Why to report/importance	The course made me aware why it is important to report. It felt good to do so and contribute to better information regarding available information.
		I think it is important everyone reports, because by doing so, some drugs would not be prescribed so easily and furthermore we could better inform patients regarding highly prevalent side-effects.
Pharmacovigilance Centre Lareb		
	Getting to know Lareb and its role as knowledge centre	To easily check at Lareb whether an encountered ADR has been reported earlier.
		I did not know Lareb before this course. Now I have encountered Lareb frequently and I am also more aware of adverse drug reactions and to report them.
	Process of assessing the reported ADR	The quick handling of the report by an employee of Lareb.
		How Lareb proceeds with a report.
	Getting feedback (letter)	I enjoyed the feedback letter, I really felt my ADR-report was meaningful.
		The extensive response/feedback letter from Lareb was very educational.

* Indicate correct answers

## Discussion

Specialist oncology nurses are capable of reporting ADRs, as evidenced by their good clinical documentation of the ICSRs and by the relevance of their reports. The reporting assignment yielded valuable, relevant and well-documented ADR reports for pharmacovigilance. The nurses were ready for their role in pharmacovigilance practice, had positive attitudes/intentions and had adequate skills/knowledge about pharmacovigilance and ADR reporting after they had completed the prescribing qualification course.

The value of ADR reports made by health professionals receiving further training has not been studied earlier with a validated instrument. An earlier study of nurses reported that only 48% (95% CI 42.4–53.7) of ADR forms were complete in all relevant aspects (Ranganathan et al. 2003). Furthermore, healthcare professionals scored a mean of 78% on the ClinDoc instruments’ pilot study (Rolfes et al. 2016b). The levels of completeness of both referenced studies are considerably lower compared to the mean 89% ClinDoc score in the present study. The high scores for clinical documentation of the ICSRs in the present study show that specialist oncology nurses are highly capable of providing relevant and appropriate information in their ADR reports, even in a training situation.

Most of the reported ADRs were very relevant for pharmacovigilance, most frequently because the ADR was serious or the suspect drug was listed by EMA for additional monitoring. In 39% of the ICSRs, the ADR was serious according to CIOMS criteria, a significantly higher percentage than reported in three earlier studies of ADR reporting by nurses. In a study by Ranganatan, nurses reported a higher proportion of serious suspected ADRs than general practitioners and hospital physicians (13.5 versus 12.9 and 9.1%, respectively) (Ranganathan et al. 2003). The opposite was found in a French study in 1995, which found that doctors reported more (suspected) serious ADRs than nurses (19 versus 10%, respectively) (Sacilotto et al. 1995). In a previous study in the Netherlands, 25% of ADRs reported by health professional were considered serious (Rolfes et al. 2015). The higher number of serious ADRs in our present study is probably due to the high frequency of severe and serious ADRs to the cytostatic agents used in oncology. Although specialist oncology nurses are only permitted to prescribe a small set of supportive drugs, they apparently felt responsible for reporting ADRs to the cytostatic agents themselves.

The specialist oncology nurses had positive attitudes/intentions and adequate skills/knowledge about pharmacovigilance and ADR reporting. Almost all nurses intended to report serious ADRs in the future (mean 6.6 SD 0.6). The specialist oncology nurses had higher scores in this respect than medical students (mean 6.2; SD 1.0) (Schutte et al. 2017c), pharmacists (mean 5.2; SD 1.5) (Gavaza et al. 2011) and pharmacy students (mean 5.9; SD 1.5) (Gavaza and Bui 2012), who were all assessed with the same questionnaire. These results are possibly related to the nurses’ expectations regarding the relevance, benefits and costs of reporting. Although the nurses expected reporting to be somewhat time consuming (mean 4.5 SD 1.6 on 7-point Likert scale), pharmacists thought that it would be more time consuming (mean 5.1 SD 1.6) (Gavaza et al. 2011). More nurses than pharmacists considered that reporting is “personally beneficial” (mean 6.2 SD 0.6 versus mean 5.0 SD 1.6, respectively) (Gavaza et al. 2011). Furthermore, their skills and knowledge in pharmacovigilance were considerably better than those of final-year medical students in the Netherlands (scores of 75 versus 68% for basic pharmacovigilance knowledge, respectively) (Schutte et al. 2017c). All but one of the nurses knew where and what was necessary to report for a good ADR report. This was far better than final-year medical students, of whom 78% knew where they should report an ADR and 33% knew what was necessary for a good ADR report. The ADR-reporting assignment and plenary discussion were considered useful by the nurses, and they commented that it changed how they dealt with ADRs. All these findings are important as they potentially influence the willingness and likelihood of these nurses reporting serious ADRs in the future.

The main limitation of this study is its relative small sample size and design—there was no pre-participation measurement or control group. Moreover, the population mainly consisted of experienced female nurses. Furthermore, the educational setting might have biased results, since the nurses may have put more effort into the assignment than they would do in their usual workplace settings. Taken these limitations into account, this study showed the value of an ADR reporting assignment, as it offered oncology nurses valuable experience and training while providing valuable and relevant ADR reports for pharmacovigilance. Earlier studies have demonstrated comparable positive outcomes of giving learners legitimate roles in real (pharmacotherapy) problems of real patients (Dekker et al. 2015; Schutte et al. 2017a; Schutte et al. 2017b).

Given the positive results of the ADR reporting assignment in this population, future research should focus on optimization of pharmacovigilance education for health professionals, especially assignments that are grounded in real practice. Such research should study the long-term effects of educational interventions and whether less experienced health professionals are also capable of contributing to pharmacovigilance while performing practical assignments in real practice, as we demonstrated in this study. To conclude, the adverse drug reaction reporting assignment yielded valuable, relevant and well-documented ADR reports for pharmacovigilance. In addition, this assignment was considered educational by the specialist oncology nurses. The participating nurses had positive attitudes/intentions and had adequate skills/knowledge about pharmacovigilance and ADR reporting after they had completed the prescribing qualification course.

## Electronic supplementary material

ESM 1 (DOCX 57 kb)
