# Supplementary material for: The adverse drug reaction reporting assignment for specialist oncology nurses: a preliminary evaluation of quality, relevance and educational value in a prospective cohort study
Source: Naunyn Schmiedebergs Arch Pharmacol. 2017 Oct 23;391(1):17–26. doi: 10.1007/s00210-017-1430-z (PMC5748417; doi:10.1007/s00210-017-1430-z)
Supplement: Supplementary file 1 — (DOCX 57 kb) [file 210_2017_1430_MOESM1_ESM.docx]

**Appendix_A**

**The adverse drug reaction reporting assignment for specialist oncology nurses: A preliminary evaluation of quality, relevance and educational value in a prospective cohort study**

Tim Schutte^1,2^, Rike van Eekeren^3,4,5^, Milan Richir^1,2^, Jojanneke van Staveren^6^, Eugène van Puijenbroek^3,4,5^, Jelle Tichelaar^1,2^, Michiel van Agtmael^1,2^

1 Department of Internal Medicine, Pharmacotherapy Section, VU University Medical Center, Amsterdam, the Netherlands

2 RECIPE (Research & Expertise Center In Pharmacotherapy Education), Amsterdam, the Netherlands

3 The Netherlands Pharmacovigilance Centre Lareb, Den Bosch, the Netherlands

4 Department of Pharmacy, Pharmacotherapy and Pharmaceutical Care, University of Groningen, Groningen, the Netherlands

5 WHO Collaborating Centre for Pharmacovigilance in Education and Patient Reporting

6 Amstel Academy, VU University Medical Center, Amsterdam, the Netherlands

**Corresponding author:** Tim Schutte, [t.schutte@vumc.nl](mailto:t.schutte@vumc.nl)

Department of Internal Medicine, Pharmacotherapy Section,

De Boelelaan 1117 1081 HZ, Amsterdam, The Netherlands

Room ZH4A50, Phone +31 20 4448090

| *Question* | | *Answer option* |
| --- | --- | --- |
| *1. If you suspect a patient to have an adverse drug reaction, how would you act?* | | *Open question* |
| *2. I know where to report an ADR (in the Netherlands)?* | | *yes / no (if yes, please specify)* |
| *3. Have you reported an ADR to the Dutch pharmacovigilance centre Lareb before you enrolled in this module?* | | *yes / no (if yes, please specify)* |
| *4. Has the subject side effects been covered in any form during your training as (specialist) nurse?* | | *yes / no (if yes, please specify)* |
| *5. Has the subject ADR reporting been covered in any form during your training as (specialist) nurse?* | | *yes / no* |
| *6. Can you indicate what you learned in this ADR-reporting assignment* | | *Open* |
| *7. Statements concerning the value of the reporting assignment*  *The ADR-reporting assignment…* | | *5 point Likert scale (1: strongly disagree – 5 strongly agree)* |
|  | *… was useful* |  |
|  | *… suited well within my practice* |  |
|  | *… improved attention for medication and patient safety* |  |
|  | *… is more instructive than learning with fictive casuistry* |  |
|  | *… cost a lot of time to do* |  |
|  | *… did change my practice in dealing with ADRs* |  |
| *8. Statements concerning the value of the plenary discussion of the reporting assignment*  *The ADR-reporting assignment discussion…* | | *5 point Likert scale (1: strongly disagree – 5 strongly agree)* |
|  | *… was useful* |  |
|  | *… is more instructive than learning with fictive casuistry* |  |
| *9. How likely do you think the following outcomes will be if you report an ADR to the Dutch Pharmacovigilance Centre Lareb?* | | *7 point Likert scale (1: extremely unlikely – 7 extremely likely)* |
|  | *It contributes to the safe use of medicines.* |  |
|  | *Improves patient safety* |  |
|  | *Educates others about drug risks* |  |
|  | *Personally beneficial* |  |
|  | *Time consuming to report* |  |
|  | *Disrupts the normal workflow* |  |
|  | *Increases risk of malpractice* |  |
|  | *Breaks trust with patients* |  |
| *10. What is your opinion regarding the following statements?* | | *7 point Likert scale (1: extremely unlikely – 7 extremely likely)* |
|  | *Pharmacovigilance should be included as a core topic in the curriculum of all prescribers* |  |
|  | *Pharmacovigilance is well covered (up to now) in my curriculum* |  |
|  | *I do not know how I could report an ADR to the relevant authorities* |  |
|  | *Students can report ADRs during their clerk/internships* |  |
|  | *Reporting known ADRs makes no significant contribution to the reporting system.* |  |
|  | *With my present knowledge, I am very well prepared to report any ADRs in my future practice.* |  |
|  | *I believe that doctors are one of the most important healthcare professionals to report ADRs* |  |
|  | *I believe that pharmacists are one of the most important healthcare professionals to report ADRs* |  |
|  | *I believe that (specialist) nurses are one of the most important healthcare professionals to report ADRs* |  |
|  | *I believe serious and unexpected reactions that are not fatal or life-threatening during clinical trials should not be reported.* |  |
| *11. Could you indicate how likely it is you will report an ADR to the Dutch Pharmacovigilance Centre Lareb in the following situations:* | | *7 point Likert scale (1: extremely unlikely – 7 extremely likely)* |
|  | *I intend to report serious ADRs that I will encounter to the competent authority.* |  |
|  | *I intend to report unknown ADRs that I will encounter to the competent authority.* |  |
|  | *I intend to report all ADRs that I will encounter to the competent authority.* |  |
| *12. I know which essential information is needed for a qualitative good ADR report* | | *yes / no (if yes, please specify)* |
| *13. What are the correct answers to the following statements?* | | *yes / no* |
|  | *All ADRs, irrespective of severity, must be reported.* |  |
|  | *Doctors should report serious ADRs even if uncertain that product caused the event.* |  |
|  | *Doctors should report serious ADRs even if do not have all details of event.* |  |
|  | *All serious ADRs are known before a drug is marketed.* |  |
|  | *Lareb does not disclose ADR reporter’s identity.* |  |
|  | *One can report ADRs anonymously to Lareb.* |  |
|  | *Adverse experiences with cosmetics and special nutritional products may be reported to Lareb* |  |
|  | *Adverse experiences with natural or homeopathic products may be reported to Lareb* |  |
|  | *Adverse experiences with vaccines may be reported to Lareb* |  |
|  | *One case reported by a doctor does not contribute much to knowledge about drug risks.* |  |
|  | *I have adequate knowledge of ADR reporting* |  |
|  | *Patients can report ADRs independent from a healthcare professional.* |  |

**Table 1:** E-survey questions

|  | **TYPE OF DRUG** | **ADR (SYSTEM ORGAN CLASS)** | **ClinDoc**  **SCORE (%)** | **DESCRIPTION** | **SERIOUS** | **ADDITIONAL MONITORING** | **ADR NOT LABELED** | **OFF LABEL USE** | **ADR CAUSED WITHDRAWAL OF ONCOLYTIC THERAPY** |
| --- | --- | --- | --- | --- | --- | --- | --- | --- | --- |
| 1 | Cytostatic | Respiratory, thoracic and mediastinal disorders | 94% | well | 1-H | 0 | 0 | 0 | 0 |
| 2 | Cytostatic | Eye disorders | 81% | well | 0 | 0 | 0 | 0 | 0 |
| 3 | Cytostatic | Reproductive system and breast disorders | 75% | well | 0 | 0 | 1 | 0 | 0 |
| 4 | Cytostatic | Gastrointestinal disorders/ Skin and subcutaneous tissue disorders | 89% | well | 0 | 1 | 0 | 0 | 0 |
| 5 | Cytostatic | Skin and subcutaneous tissue disorders | 100% | well | 0 | 0 | 0 | 0 | 0 |
| 6 | Supporting | Respiratory, thoracic and mediastinal disorders | 77% | well | 0 | 0 | 0 | 0 | 0 |
| 7 | Cytostatic | Skin and subcutaneous tissue disorders | 92% | well | 1-H | 0 | 0 | 0 | 0 |
| 8 | Supporting | Psychiatric disorders | 100% | well | 1-H | 0 | 1 | 1 | 0 |
| 9 | Cytostatic | Respiratory, thoracic and mediastinal disorders / Gastrointestinal disorders / General disorders and administration site conditions | 89% | well | 0 | 0 | 1 | 0 | 0 |
| 10 | Supporting | Gastrointestinal disorders | 94% | well | 0 | 0 | 0 | 1 | 0 |
| 11 | Diagnostic | Immune system disorders | 94% | well | 1-H | 0 | 0 | 0 | 0 |
| 12 | Cytostatic | Gastrointestinal disorders/ Skin and subcutaneous tissue disorders | 92% | well | 0 | 0 | 0 | 0 | 0 |
| 13 | Cytostatic | Skin and subcutaneous tissue disorders | 100% | well | 0 | 0 | 0 | 0 | 0 |
| 14 | Cytostatic | Gastrointestinal disorders | 94% | well | 1-D | 0 | 0 | 0 | 0 |
| 15 | Cytostatic | Respiratory, thoracic and mediastinal disorders | 92% | well | 1-H | 0 | 0 | 0 | 0 |
| 16 | Cytostatic | Psychiatric disorders | 61% | moderate | 0 | 1 | 1 | 0 | 0 |
| 17 | Cytostatic | Eye disorders | 100% | well | 0 | 0 | 0 | 0 | 0 |
| 18 | Cytostatic | Gastrointestinal disorders | 100% | well | 0 | 1 | 0 | 0 | 0 |
| 19 | Cytostatic | Skin and subcutaneous tissue disorders | 78% | well | 0 | 0 | 0 | 0 | 0 |
| 20 | Other treatment* | Blood and lymphatic system disorders | 83% | well | 1-L | 0 | 0 | 0 | 0 |
| 21 | Cytostatic | Skin and subcutaneous tissue disorders / Nervous system disorders | 83% | well | 0 | 0 | 0 | 0 | 1 |
| 22 | Other treatment* | Hepatobiliary disorders | 79% | well | 1-O | 0 | 1 | 0 | 1 |
| 23 | Cytostatic | Cardiac disorders / Renal and urinary disorders | 100% | well | 1-H | 0 | 1 | 0 | 0 |
| 24 | Supporting | Gastrointestinal disorders / Nervous system disorders | 100% | well | 0 | 0 | 0 | 0 | 0 |
| 25 | Cytostatic | Nervous system disorders | 89% | well | 0 | 1 | 1 | 0 | 0 |
| 26 | Supporting | Gastrointestinal disorders | 81% | well | 0 | 0 | 0 | 0 | 0 |
| 27 | Cytostatic | Gastrointestinal disorders | 89% | well | 1-H | 0 | 0 | 0 | 0 |
| 28 | Supporting | Musculoskeletal and connective tissue disorders | 92% | well | 0 | 1 | 0 | 0 | 0 |
| 29 | Cytostatic | Gastrointestinal disorders / Drug interaction | 75% | well | 1-H | 0 | 0 | 0 | 0 |
| 30 | Supporting | Eye disorders | 100% | well | 1-H | 0 | 0 | 0 | 0 |
| 31 | Cytostatic | Skin and subcutaneous tissue disorders | 83% | well | 1-H | 0 | 0 | 0 | 1 |
| 32 | Cytostatic | Skin and subcutaneous tissue disorders | 100% | well | 0 | 0 | 0 | 0 | 0 |
| 33 | Cytostatic | Skin and subcutaneous tissue disorders | 81% | well | 0 | 0 | 0 | 0 | 1 |

**Table 2:** Information on reported ICSRs by student specialised nurses in oncology, Jan-March 2016. 1= true, 0 false, SERIOUS: According to CIOMS criteria, 1-H Hospitalization, 1-L: life threatening, 1-D: death, 1-O: other; * Other treatment, other drug, not classified as cytostatic or in support of present cytostatic therapy.
